# Overexpression of a Sucrose Synthase Gene Indirectly Improves Cotton Fiber Quality Through Sucrose Cleavage

**DOI:** 10.3389/fpls.2020.476251

**Published:** 2020-11-12

**Authors:** Mukhtar Ahmed, Adnan Iqbal, Ayesha Latif, Salah ud Din, Muhammad Bilal Sarwar, Xuede Wang, Abdul Qayyum Rao, Tayyab Husnain, Ahmad Ali Shahid

**Affiliations:** ^1^Centre of Excellence in Molecular Biology (CEMB), University of the Punjab, Lahore, Pakistan; ^2^Institute of Crop Sciences, College of Agriculture and Biotechnology, Zhejiang University, Hangzhou, China; ^3^Department of Higher Education, Government Boys College Sokasan, Azad Jammu and Kashmir, Pakistan; ^4^Institute of Molecular Biology and Biotechnology (IMBB), Center for Research in Molecular Medicine (CRM), University of Lahore, Lahore, Pakistan

**Keywords:** cotton fiber, sucrose synthase gene, *Agrobacterium*-mediated transformation, overexpression, genetic modification

## Abstract

The study aims to improve fiber traits of local cotton cultivar through genetic transformation of sucrose synthase (*SuS*) gene in cotton. Sucrose synthase (SuS) is an important factor that is involved in the conversion of sucrose to fructose and UDP-glucose, which are essential for the synthesis of cell wall cellulose. In the current study, we expressed a synthetic *SuS* gene in cotton plants under the control of a CaMV35S promoter. Amplification of an 813-bp fragment using gene-specific primers confirmed the successful introduction of *SuS* gene into the genome of cotton variety CEMB-00. High *SuS* mRNA expression was observed in two transgenic cotton plants, MA0023 and MA0034, when compared to the expression in two other transgenic cotton plants, MA0035 and MA0038. Experiments showed that *SuS* mRNA expression was positively correlated with SuS activity at the vegetative (54%) and reproductive stages (40%). Furthermore, location of transgene was found to be at chromosome no. 9 in the form of single insertion, while no signal was evident in non-transgenic control cotton plant when evaluated through *fluorescent in situ hybridization* and karyotyping analysis. Fiber analyses of the transgenic cotton plants showed increases of 11.7% fiber length, 18.65% fiber strength, and up to 5% cellulose contents. An improvement in the micronaire value of 4.21 was also observed in the MA0038 transgenic cotton line. Scanning electron microscopy (SEM) revealed that the fibers of the *SuS* transgenic cotton plants were highly spiral with a greater number of twists per unit length than the fibers of the non-transgenic control plants. These results determined that *SuS* gene expression influenced cotton fiber structure and quality, suggesting that *SuS gene* has great potential for cotton fiber quality improvement.

## Introduction

Cotton, being the chief source of natural fiber, contributes 1.8% of the gross domestic production (GDP) in the form of foreign earnings from the sale of textile products across the globe. Pakistan ranks fourth in cotton production and is third in both export and consumption (Economic Survey of Pakistan, 2016–17; Cotistics, 2018). Maturity and fiber length are important characteristics in the textile sector, which ultimately impact spinning (Kelly et al., 2019). The immature fibers are too delicate and form neps during spinning, while overly mature fibers have thick cell walls and yield coarse, thick yarns that are not favored by users (Chee et al., 2005; Kim et al., 2013). In addition, short-length fibers (fuzz) cannot be twisted multiple times during spinning; therefore, long fibers are preferred because they twist more easily. The import of long fibers in Pakistan for the textile industry has been estimated at ∼55,000 tons (Ahmed et al., 2018b). The industrial requirement of high-quality natural fiber demands for its quality improvement. With the advancement of technology and emergence of new tools for uncovering the genome at gene level, along with development of technologies like gene transformation and genome engineering offers new ways to improve cotton fiber quality by foreign gene insertion (Campbell et al., 2018). The transgene expression will not only help in enhancement of trait but also shorten the breeding time (Li et al., 2009). Keeping in mind the complexity of cotton fiber quality perfection due to its multifactorial nature, one of the important fiber traits is linked with cellulose pathway. This trait was supposed to be introduced into cotton for fiber improvement as claimed previously through transformation of silkworm fibroin gene (Zhang et al., 2004; Li et al., 2009). Similarly, another study also claimed the improvement of fiber length and strength of cotton through transformation of *acsA* and *acsB* genes (Li et al., 2004).

Cotton fiber quality, like staple length and maturity, is influenced by various factors at different developmental stages. During development, leaf subtending to a cotton boll is a chief source of carbohydrates that accounts for 60–80% of the plant’s total requirement. The role of subtending leaf and changes in sucrose metabolism is very important in determining the cotton yield, number of bolls, and fiber development (Liu et al., 2013). Sucrose is the ultimate product of photosynthesis in source tissues (leaf) that is translocatable in plants and need to be cleaved into its component hexoses in the sink (cotton boll/fruit) before further biochemical reactions. Developing cotton bolls and seeds serve as an active sink for import of assimilates through phloem unloading. The imported assimilates are directed toward seed coat epidermis for fiber cell development, cellulose synthesis, and toward filial tissue for embryo growth (Ruan, 2005).

Cotton fiber development consists of four distinct but overlapping stages: initiation, elongation, maturation, and a secondary wall synthesis, which are all controlled by numerous genes, transcription factors, and phytohormones (Lee et al., 2007; Zhang et al., 2017; Xiao et al., 2019). Sugars act as signaling molecules that influence the function of promoters and transcription factors (Ruan, 2012). Sugar, together with cellulose, is crucial for lint length and strength in cotton at the time of secondary cell wall synthesis (Murray et al., 2001). Cellulose synthesis starts at the end of primary wall formation, and its accumulation is pivotal at the time of secondary wall synthesis. Secondary cell wall leads to an increase in cellulose synthesis and maturation of lint fibers through dehydration and thus is crucial for determination of cotton fiber quality (Abidi et al., 2010; Pang et al., 2010).

In-seed kernel sucrose metabolism is mainly governed by three enzymes: sucrose phosphatase synthase (SPS), invertase (INV), and sucrose synthase (SuS). SPS synthesizes sucrose from UDP-glucose (UDPG) after several interconversions. Sucrose synthesis can occur both in source and sink cells by successive action of SPS and sucrose phosphate phosphatase (SPP). SPS forms sucrose-P from F-6-P (fructose-6-phoshate) and UDPG, and reaction moves in synthetic direction by fast conversion of sucrose-P to sucrose by SPP (Botha and Black, 2000). Invertases catalyze irreversible sucrose cleavage into glucose and fructose. The sucrose synthase (SuS) plays a significant role in fiber development by providing component hexoses for cellulose synthase. These hexoses maintain the osmotic potential required to build turgor pressure during fiber elongation and cellulose synthesis (Weschke et al., 2003; Wang and Ruan, 2013; Ahmed et al., 2018a).

SuS exists in two forms, membrane-bound SuS (M-SuS), which is proposed to be involved in cell wall synthesis by providing carbon to cellulose synthase for cellulose synthesis (Amor et al., 1995), and cytoplasmic SuS (C-SuS), which is involved in the reversible breakdown of sucrose. Although the reaction is reversable, the cleavage of sucrose and UDP into fructose and UDP-glucose (UDPG) is favored (Gou et al., 2007; Kleczkowski et al., 2010; Brill et al., 2011):

Sucrose + UDP UDP-glucose + fructose

UDPG provides glucose as a substrate for enzymes in a number of glycosylation reactions. UDPG is also essential for the production of sucrose in the cytoplasm and for the formation of cellulose and callose in apoplasts (Stein and Granot, 2019). SuS built a putative complex with cellulose synthase located on the plasma membrane and directs carbon from sucrose to cellulose (Amor et al., 1995). The secondary wall of the cotton fibers that are used for the production of apparel contains 95% cellulose (Haigler et al., 2001).

The SuS activity at early stages of cotton fiber development (0–5 days post anthesis) determined the fate of ovule epidermal cells to form fibers. Reduced SuS activity at early stage of fiber initiation led to fiberless seed phenotype (Ruan et al., 2005; Ahmed et al., 2019). Beside this, potato SuS gene overexpression in cotton enhanced fiber production (Xu et al., 2012). It seems reasonable to elucidate the role of SuS in improvement of fiber length, strength, and micronaire/smoothness. So far, cotton fiber quality has not been successfully addressed through conventional means due to species compatibility. However, biotechnological techniques in combination with traditional breeding may be useful to sort out the serious issue of cotton fiber quality (Constable et al., 2015).

The improvement in fiber length through expression of expansin gene of *Calotropis procera* was already reported in our previous studies (Bajwa et al., 2013; Akhtar et al., 2014). Similarly, expression of GhActin 1, HOX3, GhWlim5, and PIP gene resulted in improved fiber length, and micronaire value (unpublished data) has also been investigated. In the current study, we attempted to transform a codon-optimized *SuS* gene into a non-transgenic, local cotton variety by using *Agrobacterium*-mediated transformation protocol. We found positive impact of *SuS* gene expression in cotton fiber quality improvement. Our results implied that elevated levels of SuS activity increased the supply of carbohydrates (glucose and fructose) to sink tissue (fibers) by catalyzing the cleavage of sucrose, which ultimately increased fiber elongation by increasing turgor pressure. This increased supply of carbohydrates also improves fiber cellulose contents and results in smoothening of cotton fiber surface. The elevation of SuS activity in leaves resulted in leaf expansion through increase in leaf surface area, plant height, number of bolls, and boll weight. The growth and fiber development are the ultimate result of increase in supply of photo assimilates from source to sink tissues. From the results, it can be envisioned that SuS is one of the important factors involved in fiber development and can provide an opportunity to the breeders for utilization of germplasm with other fiber-related factors in combination to meet the requirements of local textile industry. Having the background knowledge of *SuS* gene activity at different stages of fiber development, length and strength of transgenic cotton harboring *SuS* gene is expected to be improved.

## Materials and Methods

### Plant Material

Non-transgenic cotton variety CEMB-00 was selected for SuS gene transformation as its fiber characteristics are below the optimal range. Seeds of cotton variety CEMB-00 were collected from CEMB Research Station Multan (30° 5′ 0″ N, 71° 40′ 0″ E) Punjab, Pakistan.

### SuS Transgene Construct and Plant Transformation

**FIGURE 1:**
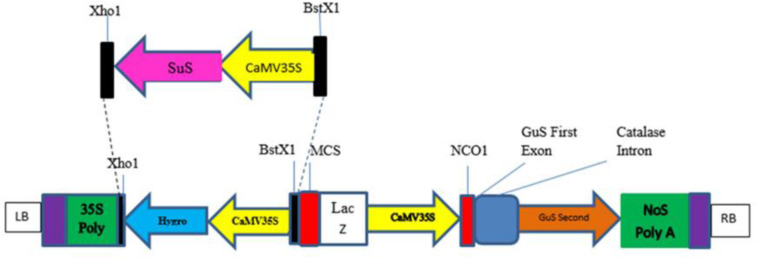
Schematic diagram of a CaMV35S-sucrose synthase (SuS) construct.

The sequence of *Zea mays* sucrose synthase 1 gene (Gene Bank Accession number NM_001111853.1) was retrieved from the National Centre for Biotechnology Information GenBank database^1^ (Pruitt et al., 2006). The sequence was optimized by replacing codons predicted to be less frequently used in cotton with more favored codons without altering the amino acid sequence (Perlak et al., 1991; Owczarzy et al., 2008; Latif et al., 2015) with the help of codon usage databases^2, 3^. The codon-optimized sequence was checked for structural and functional efficiency using some bioinformatics tools available on SIB Bioinformatics Resource Portal^4^ (Gasteiger et al., 2003; Arnold et al., 2009). A codon-optimized *SuS* gene under the control of the CaMV35S promoter with B*st*XI and X*ho*I restriction sites (Figure 1) was chemically synthesized and cloned into the pUC57 vector, which contains the ampicillin selection gene by Bio Basic Inc.,^5^ and was received in a lyophilized form. The plasmid construct harboring the *SuS* gene (pUC_SuS) was confirmed by restriction digestion with *Bst*X1 and *Xho*1. Then, the excised fragment was ligated into the plant expression vector pCAMBIA1301 using the same enzymes. The resulting plasmid, pCAMBIA1301_SuS, was transformed into *Agrobacterium* LBA4404 competent cells. Cloning of the *SuS* fragment into the plant expression vector was confirmed by restriction digestion and PCR using the detection primers: SuS F 5′-CTTGAGAAATTCTTGGGTACTATCC-3′ and SuS R 5′-CTCGGTGTAAGGGAAATAAATAGAC-3′. *Agrobacterium* cells transformed with pCAMBIA1301_SuS were grown in Luria Bertani (LB) medium containing rifampicin and kanamycin (50 mg/ml). Then, the cells were harvested and suspended in 10 ml of Murashig and Skoog (MS) broth (Murashige and Skoog, 1962). The cotton variety CEMB00 was transformed with pCAMBIA_SuS by the *Agrobacterium* cells using a previously reported method (Rao et al., 2009; 2011). Transformation efficiency was determined by counting the number of plants that survived after 4–6 weeks on MS medium compared to the total number of embryos cocultivated in all experiments (Satyavathi et al., 2002; Rao et al., 2011). After 1 month, plants were transferred to soil composed of equal proportions of clay, sand, and peat moss (1:1:1) and kept for acclimatization.

### Molecular Analysis of Putative Transgenic Cotton Plants

#### Histochemical Analysis of GUS Expression

A histochemical GUS assay was performed using leaves from potted plantlets. The leaves were placed in GUS solution in 50-ml falcon tubes and incubated at 37°C overnight. Then, the samples were observed for the development of a blue color, indicative of GUS activity.

#### Genomic DNA Isolation and PCR

Total genomic DNA was extracted and purified from the leaves of cotton plants using the Cetyltrimethylammonium bromide (CTAB) method (Doyle and Doyle, 1990). The quality of the extracted DNA was evaluated by separation on 0.8% agarose gel and staining with 0.5–1 μg ml^–1^ ethidium bromide, and the purity was confirmed using a Nanodrop ND-1000 spectrophotometer. The *SuS* gene was amplified by PCR using the gene-specific primers SuS F and SuS R. The PCR mixture was prepared as follows: 2 μl of 10 × PCR buffer, 2 μl of 1 mM dNTPs, 2.5 U of Taq polymerase, 2 μl of 10 mM primer 1, 2 μl of 10 mM primer 2, 1.5 μl of DNA template (∼25 ng), and water to a final volume of 20 μl. The PCR was performed using the following cycling conditions: initial denaturation at 95*°*C for 4 min, followed by 35 cycles of 95*°*C for 30 s, 55*°*C for 45 s, and 72*°*C for 45 s, with a final extension at 72*°*C for 7 min.

#### RNA Isolation and Quantitative Real Time-PCR (qRT-PCR)

RNA was isolated from fiber samples using an already reported method (Jaakola et al., 2001; Sarwar et al., 2019). Extracted RNA was treated with DNase1 (1 μg), and then the *SuS* transcript expression was determined by qRT-PCR (BioRad iCycler iQ5). *SuS* expression levels were normalized with *GAPDH*. For the first-strand cDNA synthesis, 1 μg of RNA template and the cDNA synthesis kit (Fermentas cat#1622) were used. cDNA synthesis was performed at 42*°*C for 30 min, followed by incubation at 70*°*C for 10 min. Then, 2 μl of the cDNA was used in a 25-μl reaction. The gene-specific primers SuS F, SuS R, GAPDH F (5′- AGGAAGAGCTGCTTCGTTCA-3′), and GAPDH R (5′- CCGCCTTAATAGCAGCTTTG-3′) were used to analyze mRNA transcript expression.

RT-PCR was performed using SYBR Green PCR master mix (Fermentas) according to the manufacturer’s instructions. The mixture was prepared as follows: 7.5 μl of 2 × SYBR Green RT-PCR Master Mix, 1 μl of each primer (10 pM each), 0.5 μl of cDNA template, and RNase-free water to a final volume of 15 μl. After thorough mixing, the mixture was kept on ice. Relative gene expression was analyzed with the Relative Expression Software Tool (REST) from Qiagen (REST.gene-quantification.info).

#### Fluorescence *in situ* Hybridization (FISH)

Plants showing high expression of SuS were subjected to FISH for determination of copy number following the procedure published in earlier studies (Ali et al., 2016; Yasmeen et al., 2016). Probe labeling was done by Label ITFISH^®^ labeling kit CY^®^3 (MIRUS cat # 6510) following the manufacturer’s instructions. Cotton seeds were germinated, and root tips were selected for chromosome preparation. Chromosomes were hybridized with specific probe. Transgene copy number was estimated by directly visualizing the fluorescent signals present on the transgenic cotton metaphase chromosome spread after *in situ* hybridization.

#### Determination of SuS Activity and Sucrose and Total Soluble Sugar Contents

Preparation of crude enzyme extract: SuS activity was determined in tissues from the vegetative (subtending leaf) and reproductive (fibers) stages. Leaf and fiber samples were collected on different days post anthesis (DPA). SuS activity was measured at 8, 15, and 20 DPA. Cotton fibers and subtending leaf (0.5 g each) were taken, 2 ml of 50 mM phosphate buffer pH 8.0 (2.56 ml 0.6 mol/L Na_2_HPO_4_, 1.64 ml 0.2 mol/L Na- EDTA, 0.75 ml 0.6 mol/L NaH_2_PO_4_, volume raised up to 100 ml with water) was added. The mixture was homogenized by grinding in chilled pestle and mortar. The slurry was centrifuged at 5*°*C, 4,500 rpm for 20 min, and the supernatant was taken into 1 ml of ammonium sulfate precipitation. Fifty percent of saturated sediment was suspended in 0.2 ml of 50 mM phosphate buffer then poured into a dialysis bag, placed in 1,000 ml of 10 mM phosphate buffer pH 8.0 (10.2 ml of 0.6 mol/L Na_2_HPO_4_, add 6.6 ml of 0.6 mol/L NaH_2_PO_4_ and 10 ml of 0.2 mol/L EDTA-Na volume raised up to 2,000 ml with water), and kept at 0∼4*°*C for overnight dialysis with constant volume to 0.5 ml (crude enzyme extract). SuS activity was measured as described previously (Ahmed et al., 2019).

Determination of total soluble sugars: Cotton fiber samples were taken at 15, 30, and 40 DPA. Sucrose and total soluble sugar contents were measured as reported earlier (Wang et al., 2011; Ahmed et al., 2019).

#### Determination of Cellulose Contents

To measure the cellulose contents, mature fiber samples from three control and three transgenic plants of each line were collected and dried at 100°C. Then, about 0.5 g of the dried fibers was pulverized to form a fine powder and used to measure cellulose contents according to a previously reported method (Updegraff, 1969; Yuan et al., 2012; Bajwa et al., 2015). A standard curve was constructed with different concentrations of microcrystalline cellulose (Avicel). The absorbance was read at 620 nm with a spectrophotometer (Beckman Coulter DU-520).

#### Fiber Characteristics

Mature and opened cotton bolls were harvested from the midregion of plants. Bolls damaged due to insects, rain, or other stress were excluded. Fiber samples (60 g) were collected from transgenic and control cotton plants, and fiber quality assessments of length, strength, uniformity, and micronaire were performed using a High Volume Instrument (HVI) SW version 3.3.5.57 at the Fiber Technology Section of the Central Cotton Research Institute (CCRI)^6^ in Multan, Pakistan. Three samples from each line were tested, and each reported value is the mean of three biological replicates.

#### Scanning Electron Microscopy Analysis of Mature Fibers

For scanning electron microscopy (SEM) analysis, mature fibers from 30 transgenic and control cotton plants were collected, and each fiber was cut into three parts: the tip, middle, and base. Then, the samples were analyzed using a scanning electron microscope (SEM, Model SU8010; Hitachi, Japan). The screw pitch of the fiber and the distance of the fiber rotation at 360° of every sample were measured three times under SEM at 400×, 1,000×, and 4,000× magnification.

#### Phenotypic Characteristics of Transgenic Cotton Plants

Phenotypic characteristics of transgenic cotton plants, including plant height, boll number, and average boll weight and leaf surface area was determined and compared with non-transgenic control cotton plants. The height of mature plants was measured from base to the apex by using a measuring tape. Three plants from each transgenic and control line were selected. Each value was represented by the mean of three plants. The total number of bolls on each transgenic and control plants were counted. The total number of bolls was divided by the total number of plants studied for calculating the mean value. Average weight was calculated by dividing total seed cotton yield of all transgenic plants by their respective number of effective bolls. The average leaf area of transgenic and control plants (three plants from each line) was calculated using graphics software Adobe Photoshop 7.0 (Easlon and Bloom, 2014). Leaves were sampled randomly from top, mid, and base of the 60-days plants. Values represent the average of three leaves of every plant from each transgenic and control line. Digital photographs of transgenic and control plant leaves were taken with default parameters and further processed with ImageJ Software for the calculation of the total leaf area. Leaves were spread on the white surface with a known surface area (100 cm^2^ as a reference) in the center. The photographs of leaves were taken and analyzed by Adobe Photoshop. The leaf area of both known (used as reference or standard) and unknown leaf samples was calculated by computing pixel values. The surface area of the leaves was calculated using a unitary method.

### Statistical Analysis

Ordinary ANOVA (one-way analysis of variance, Dunnett’s test) and two-way ANOVA (Tukey’s test) were performed to compare the significance level between transgenic cotton lines with non-transgenic control at 95% confidence interval using GraphPad Prism (GraphPad Prism software version 5 for Windows).

## Results

### Generation and Identification of Transgenic Cotton Plants

Fiber quality of the wildtype cotton non-transgenic variety CEMB-00 used in the current study was as follows: fiber length 26.06 mm, strength 22.35 g/tex, micronaire 5.76, uniformity index % 80.00, and maturity ratio 0.8. The plant expression vector pCAMBIA1301_SuS, which harbors the *SuS* gene under the control of CaMV35S promoter, was transformed into the non-transgenic cotton variety CEMB 00 through *Agrobacterium*-mediated transformation. After root development, the plantlets were shifted to MS tubes and then to soil pots and covered with plastic bags. They were maintained in a growth room at 28 ± 2°C under a 16-h photoperiod (250–300 μmol m^–2^ s^–1^). The plants were stabilized through regular exposure to natural conditions until acclimatized. The putative transgenic cotton plants were evaluated by a histochemical GUS and PCR assay. In the GUS assay, the development of a blue color indicated the presence of GUS activity and successful *Agrobacterium*-mediated transformation into the cotton plants. In contrast, no blue color, indicating no GUS activity, appeared in the leaves of non-transformed control plants (Supplementary Figure 1A). The appearance of an 813-bp amplification product on an 0.8% agarose gel in all four transgenic cotton plants and no amplification product in non-transgenic control cotton plants confirmed the successful introduction of the *SuS* gene as shown in Supplementary Figure 1B.

A total of 9,000 embryos were transformed by the *Agrobacterium* method, and of these, only 97 grew to plantlets on MS medium. The transformation efficiency was calculated by counting the number of GUS and PCR-positive plants among the total embryos co-cultivated in the transformation experiments. The percent transformation efficiency was determined to be 1.07 (Supplementary Table 1). Finally, four of the surviving SuS transgenic cotton plants showed a normal growth pattern and also established themselves under field conditions. These plants were named MA0023, MA0034, MA0035, and MA0038. Seeds were carefully collected from the four transgenic cotton plants (MA0023, MA0034, MA0035, and MA0038) and sowed separately as different lines in the field to grow a T_1_ generation along with one line of non-transgenic cotton plants, which were used as a control (Supplementary Figure 2).

### Molecular Analysis of Transgenic Cotton Plants (T_1_ Generation)

A PCR assay was performed using genomic DNA isolated from the subtending leaves of T_1_ transgenic and non-transgenic cotton plants using internal detection primers. An 813-bp fragment of the SuS gene was detected in all the transgenic cotton plants analyzed (Supplementary Figure 3). No such fragment was amplified from the DNA of non-transgenic control cotton plants.

### Determination of Copy Number and Location of SuS Gene

**FIGURE 2:**
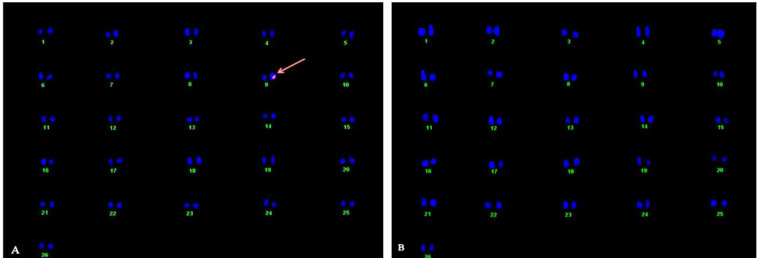
Karyotyping and location of SuS gene in the cotton genome through fluorescent *in situ* hybridization (FISH) technique. **(A)** Arrow indicates fluorescent signal at chromosome 9 in the transgenic plant. **(B)** Non-transgenic control plant.

A single pink spot on chromosome no. 9 of transgenic cotton plant line MA0023 on one chromatid and complete absence of signal in non-transgenic control determined the one copy no. in hemizygous form (Figure 2).

### Quantification of *SuS* Gene Expression in Transgenic Cotton Plants

**FIGURE 3:**
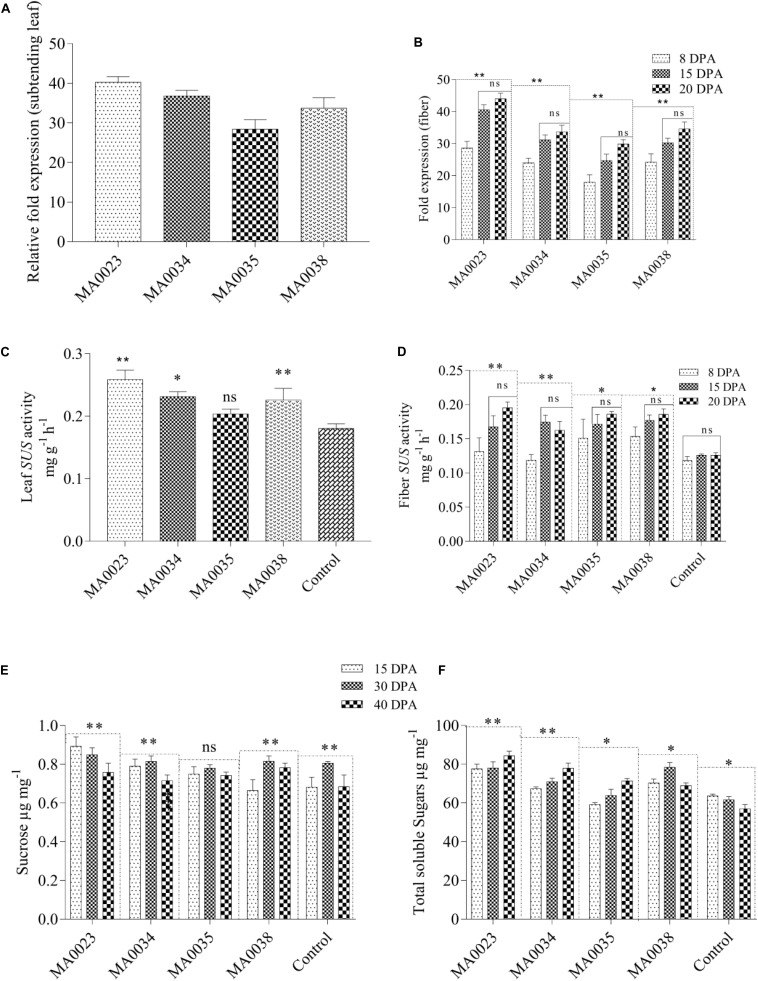
Relative expression of SuS gene mRNA **(A)** from subtending leaves of transgenic plants and **(B)** from fiber during elongation [8, 15 days post anthesis (DPA)] and early secondary wall synthesis stage (20 DPA). SuS activity in subtending leaves and fibers of transgenic and control lines. **(C)** SuS activity in leaves. **(D)** SuS activity in cotton fibers at 8, 15, and 20 DPA. Sucrose and total soluble sugars in fibers at different DPAs. **(E)** Sucrose contents in transgenic and control plants. **(F)** Total soluble sugars. Values represent the average of three replicates from each transgenic line. Asterisks indicate a significant difference between transgenic and control (^∗^*P* ≤ 0.05, ^∗∗^*P* ≤ 0.01; ns, non-significant using ANOVA).

*SuS* gene expression in subtending leaves and 8–20 DPA fibers were determined by measuring mRNA transcripts using qRT-PCR with 2 μl of cDNA from the four transgenic cotton lines. *SuS* transcript levels were higher in leaves of MA0023, MA0034, and MA0038 but low in MA0035 (Figure 3A). SuS expression in fibers of 15 and 20 DPA was high than at 8 DPA in all transgenic lines. No significant differences were observed in expression levels between 15 and 20 DPA (Figure 3B). The increased *SuS* mRNA levels were correlated with the role of SUS in fiber improvement, as the qRT-PCR analysis revealed that the *SuS* gene was upregulated beginning at the fiber elongation stage (8 DPA) and was maintained until the transition phase between the primary and secondary wall stage (20 DPA).

### Biochemical and Physiological Analyses

#### SuS Activity

Comparison of the SuS activity levels in the vegetative (subtending leaf) and reproductive (fiber development) stages of transgenic and non-transgenic control cotton plants showed significantly higher enzyme activity in the transgenic plants due to overexpression of the *SuS* gene. The SuS activity levels in the transgenic cotton lines MA0023, MA0034, and MA0038 were 0.278, 0.248, and 0.227 mg g^–1^ h^–1^, respectively, which was 54%, 37%, and 26% higher than the activity level in the non-transgenic control (0.180 mg g^–1^ h^–1^ at the vegetative stage; Figure 3C). SuS activity at the reproductive stage (15 DPA) was also increased in MA0038 to 0.177 mg g^–1^ h^–1^ (an increase of 40%). At 20 DPA, maximum activity was observed in MA0023 (0.195 mg g^–1^ h^–1^), which was an increase of 54% compared to non-transgenic control cotton plants (0.126 mg g^–1^ h^–1^). Among the transgenic cotton lines, MA0034 showed the lowest SuS activity, which was 28% higher than that in the non-transgenic control line (Figure 3D).

#### Sucrose and Total Soluble Sugars

The sucrose and total soluble sugar contents are directly proportional to SuS activity levels, which is correlated with *SuS* mRNA expression levels. In the fibers of the transgenic cotton lines, a decrease in sucrose content and an increase in total soluble sugar contents were evident at the elongation stage (15 DPA) and during secondary wall synthesis and the maturation stage (40 DPA). In the non-transgenic control cotton lines, significant variations in sucrose content were observed at different stages (Figure 3E). However, the increase in total soluble sugars from the elongation stage to the secondary wall synthesis and maturation stages was not as significant as that observed in the transgenic cotton plants (Figure 3F). Sucrose cleavage, which is catalyzed by *SuS*, was higher in the transgenic cotton plants than in the non-transgenic control cotton plants.

#### Cellulose Contents

**FIGURE 4:**
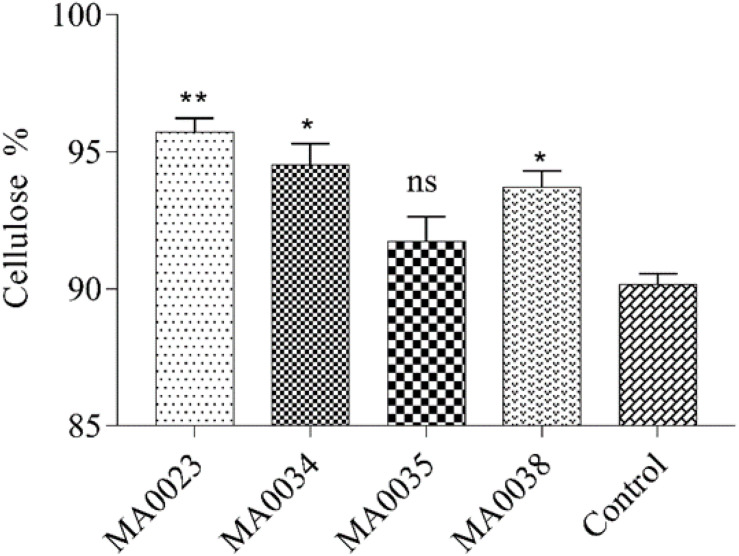
Cellulose contents in mature cotton fibers of transgenic and control plants. Values represent the average of three replicates from each transgenic line. Asterisks indicate a significant difference between transgenic and control (^∗∗^*P* ≤ 0.01; ns, non-significant using one-way ANOVA).

Analysis of the cellulose contents showed substantial increases in the transgenic cotton plants compared to the contents in the non-transgenic control cotton plants. In the transgenic cotton lines, the maximum and minimum cellulose contents were 96.2% and 92.6 % in MA0023 and MA0035, respectively. In the non-transgenic control cotton plants, the cellulose content was 90% (Figure 4). The transgenic cotton plants contained 6.8% more cellulose than the non-transgenic control plants. At the onset of maturation, a high concentration of sugar contributes to increased cellulose content in the fibers.

#### Fiber Characteristics

**FIGURE 5:**
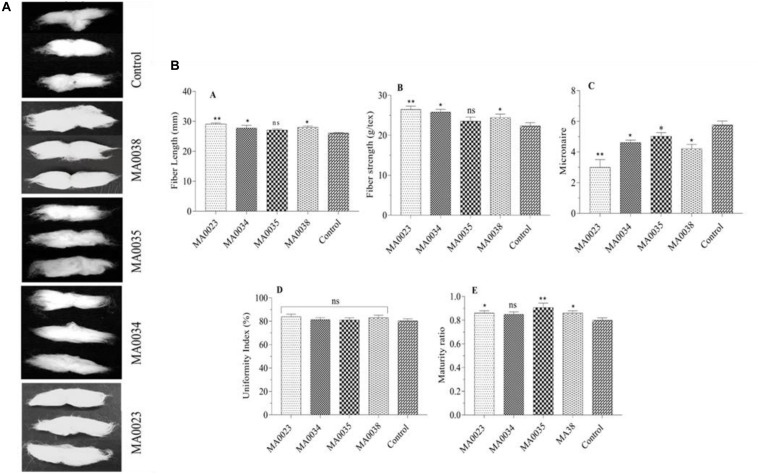
**(A)** Fiber lengths in transgenic and control plant lines. Photographs of cotton fibers in transgenic (MA0023, MA0034, MA0035, and MA0038) and control plant lines. **(B)** Fiber characteristics of transgenic and control plant lines. **(A)** Fiber strength. **(B)** Micronaire value. **(C)** Uniformity index. **(D)** Maturity ratio. **(E)** Fiber length. Values represent the average of three replicates from each transgenic and control line. Asterisks indicate a significant difference between transgenic and control (*P ≤ 0.05, **P ≤ 0.01; ns, non-significant using one-way ANOVA).

The fibers in the transgenic cotton plants were longer than the fibers in the non-transgenic control cotton plants (Figure 5). In the transgenic cotton lines MA0023, MA0038, and MA0034, the maximum fiber lengths were 29.1, 28.00, and 27.78 mm, respectively, whereas in the non-transgenic control lines, the fiber length was 26 mm. However, in one of the transgenic cotton lines, MA0035, the fiber length was 27.09 mm, which was not statistically different from that of the non-transgenic control cotton plants (Figures 5A,B). The maximum increase in fiber length, 11.7%, was observed in line MA0023.

In addition, the strength of the bundled fibers of the transgenic plants also increased to 26.46, 25.76, and 24.45 g/tex in lines MA0023, MA0034, and MA0038, respectively, compared to that of the non-transgenic control cotton plants at 22.35 g/tex (Figure 5B). Compared to the non-transgenic control cotton plants, the increases in fiber strength in the transgenic cotton lines MA0023, MA0034, and MA0038, were 18.65%, 15.25%, and 9.3%, respectively.

The transgenic cotton lines MA0034 and MA0035 showed average micronaire values of 4.6 and 5.01, respectively. The highest micronaire value, of 5.7, was measured in the non-transgenic control sample (Figure 5B). The micronaire values of MA0023 and MA0038, were found to be 3.0 and 4.21. The micronaire 4.21 in MA0038 was considered to be the optimal value as it falls within the optimal range (3.8 to 4.5), indicative of increased fineness. In the case of MA0023 and MA0035, these values are outside the optimal range, which were rated poorly, with coarse fibers depicted in the CCRI Lab reports.

Comparison of the uniformity index (UI %) between the transgenic and non-transgenic control cotton lines showed almost similar values for both groups. The transgenic cotton lines MA0023, MA0034, MA0035, and MA0038 showed UI values of 83.98%, 81.25%, 81.05%, and 83.03%, respectively, whereas the non-transgenic control cotton plant showed a UI of 80.25% (Figure 5B).

The fiber maturity values of the transgenic cotton lines MA0023, MA0034, MA0035, and MA0038 were 0.86, 0.85, 0.90, and 0.86, respectively, which were higher than the value for the non-transgenic control cotton plants, at 0.80 (Figure 5B).

**FIGURE 6:**
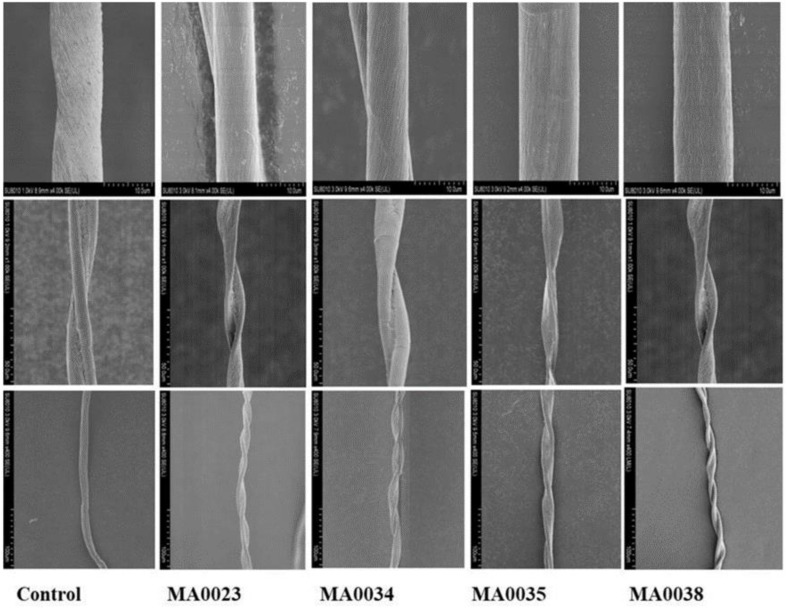
Observation of surface microstructure of transgenic and control fibers by SEM. In each row left-hand image is control fiber, while the others are transgenic fibers. **Top Row:** Surface of mature fiber at 4,000x left-hand image is control fiber, while others are transgenic fibers. The surface of control fiber is rough with wrinkles compared to the control. **Middle Row:** Surface of mature fiber at 1,000x showing twists in a single fiber. **Bottom Row:** Fibers observed at 400x; transgenic fibers highly spiraled with increased fiber twist number compared to control.

The surface microstructures of the mature fibers were observed by SEM at 400×, 1,000×, and 4,000× magnification. The analysis showed that transgenic fiber samples were highly spiral with more fiber twists per unit length when compared to the non-transgenic cotton fiber samples. SEM analysis also showed that the mature fiber surface of the transgenic cotton plants was more compact and smoother than that of the non-transgenic control cotton plants (Figure 6).

In terms of fiber length and strength, MA0023 showed better results compared to other transgenic and control cotton plants. In the case of micronaire value, MA0038 showed considerable improvement, although SEM results of MA0023 and MA0038 are not very different.

### Phenotypic Characteristics of Transgenic Cotton Plants

**FIGURE 7:**
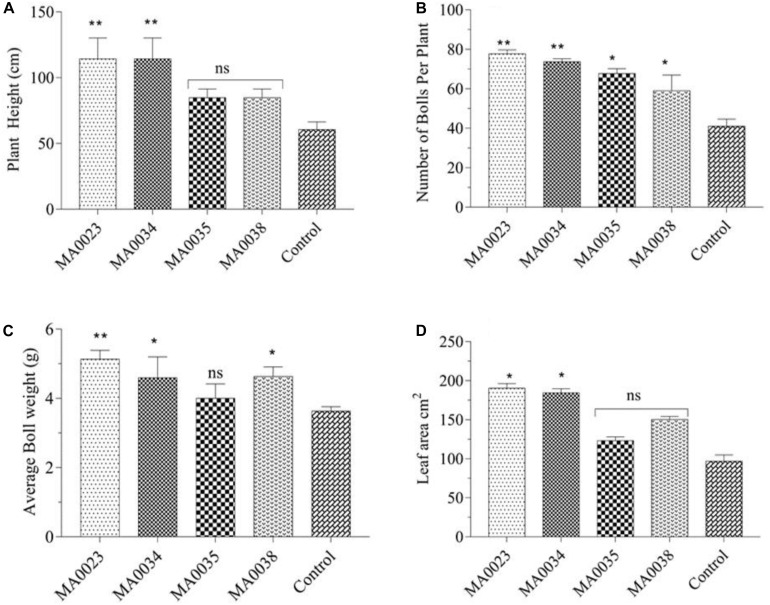
Phenotypic characteristics of transgenic and control plants. **(A)** Plant height of transgenic and control plant lines. **(B)** Number of bolls per plant line. **(C)** Average boll weight of transgenic and control plants **(D)**. Leaf area of transgenic and control cotton plants. Values represent the average of three plants from each transgenic and control line. Asterisks indicate significant difference (^∗^*P* ≤ 0.05, ^∗∗^*P* ≤ 0.01 using one-way ANOVA; ns, non-significant).

The determination of morphological characteristics was undertaken just to observe the influence of the transgenic process on plant appearance in comparison to non-transgenic control cotton plants. The obtained results indicated that plant height was increased in transgenic cotton lines compared to control. The maximum average plant height measured in transgenic cotton lines MA0023 was 114.3 cm, and the minimum height that was recorded in MA0038 was 84.75 cm. The average plant height in control non-transgenic plant line was 60.55 cm. There was an increase of 39.96% to 88.76% in height compared to non-transgenic control plants (Figure 7A). Statistical analysis showed a significant difference in MA0023 (*P* ≤ 0.01).

The total numbers of bolls were counted on each plant, and the difference between transgenic and non-transgenic control was calculated. The total number of bolls on MA0023, MA0034, MA0035, and MA0038 was found to be 77.00, 73.66, 67.60, and 59.00, respectively, while the total numbers of bolls in non-transgenic control were counted to be 41.00. Numbers of bolls in transgenic cotton plants were significantly increased compared to the non-transgenic control cotton plant (Figure 7B).

The average boll weight calculated for transgenic cotton plants was found to range from 4.00 to 5.13 g. Ten bolls from each cotton plant were randomly picked, and their average weight was calculated after weighing and drying. The lowest boll weight recorded in non-transgenic control plant was 3.63 g (Figure 7C). Comparison of average boll weight between transgenic and control is presented in Figure 7C.

Increased SuS activity was found to be correlated with leaf surface area at the initial stages of development. The leaf surface area of MA0023, MA0034, MA0035, and MA0038 was found to be 190.21, 184.39, 123.15, and 153.451 cm^2^, respectively. In the control plant, the calculated leaf area was 96.90 cm^2^ (Figure 7D).

## Discussion

Cotton is the most important cash crop in Pakistan, and its fiber is a single-cell trichome, which is considered to be an ideal system for studying the different stages of cell growth. Genetic transformation of plants is a powerful tool for trait modification, breeding, gene overexpression, and physiological and biochemical research. It is a fast, accurate, and economical alternative approach to breeding. Improvement of cotton fiber quality through genetic modification can be an excellent economical approach to achieve the desired modification in a very short time. Several genes that play key roles in cotton fiber initiation and development have been identified (Ruan, 2007; Ahmed et al., 2018b). The *SuS* gene has been reported to regulate carbohydrate interconversion and cellulose synthesis, and *SuS* overexpression and repression play a crucial role in plant growth and cotton fiber quality (Wang et al., 2011; Li et al., 2015; Stein and Granot, 2019).

In the current study, investigations were made of *SuS*-overexpressing cotton lines generated by genetic transformation. The resulting changes in sugar levels, cellulose contents, fiber length, and fiber smoothness were evaluated as reported in a previous study where overexpression of GhUGP1 in *Gossypium hirsutum* improved fiber characteristics in transgenic plants compared to the wild type (Li et al., 2015). Analysis of the mRNA expression levels of the *SuS* gene by qRT-PCR revealed a 30- to 45-fold increase in the transgenic cotton lines MA0034 and MA0023 (Figure 3), with concomitant increases in SuS activity (as measured with a spectrophotometer). A single copy number at chromosome 9 was detected through *fluorescent in situ hybridization* (FISH), while no signal was detected in non-transgenic control cotton plants (Figure 2). The results coincide with results of early researchers who determined transgene expression of Cry1ac and Cry2A along with GT gene in cotton and found a single copy number (Ali et al., 2016). This finding is consistent with a previous study in which overexpression of potato SuS in cotton under S7 promoter caused an increase in SuS activity and promoted fiber elongation (Xu et al., 2012).

Sucrose and soluble sugar levels were also correlated with the increased *SuS* gene expression levels (Figures 3E,F). The current findings are in close agreement with a little bit of deviation from previous investigations (Jiang et al., 2012; Xu et al., 2012). The overexpression of the *SuS* gene in cotton resulted in improved fiber characteristics that coincide with these studies, but improvement in vegetative growth and seed set mentioned in these reported studies may be due to difference in variety, promoter used, and climatic conditions (Jiang et al., 2012; Xu et al., 2012). In addition, in the current study, increased cellulose contents and fiber length were also observed (Figures 4, 5). It has also been reported by Jiang et al. (2012) that overexpression through *Agrobacterium* mediated transformation of SuSA1 under CaMV35S promoter resulted in increased biomass and fiber quality by increasing carbohydrate content due to elevated SuS activity in leaves and fibers. The gene SuSA1 used by Jiang et al. (2012) was taken from the cotton and was overexpressed compared to this study where the source of gene used is synthetic. While the promoter used in both studies is the same, that is, 35S, the expression of SuS in leaves and fiber at elongation stage follows almost the same trend. Antisense suppression of GhSuSA1 under the same promoter caused reverse effect on plant biomass (Jiang et al., 2012). Higher levels of sugars at fiber elongation (20 DPA) may result in increased fiber length by generating increased turgor pressure, as was evident in earlier studies (Ruan et al., 2000, 2001).

The increase in sugar content is directly proportional to the increase in turgor pressure, which results in an increase in fiber length and increase in leaf area. The elevated sugar levels enhanced the cellulose content in fiber cells by providing more carbohydrates to be polymerized into cellulose, and the increased deposition of cellulose in fiber improved the fiber characteristics. Gou et al. (2007) also reported increased levels of UDPG during secondary wall synthesis, which could be correlated with elevated SuS activity levels, as reported by Jiang et al. (2012). These results clearly demonstrate a role for SuS in fiber elongation and secondary wall biosynthesis in addition to its role in the early stages of cotton fiber development, in accordance with previous report in which this gene has been found to play an essential part in early (-2 to 5 DPA) cotton fiber development (Ahmed et al., 2019). Moreover, the *SuS* expression pattern in the transgenic cotton plants at 8, 15, and 20 DPA, positively linked with SuS activity, reflects the role of this gene in fiber elongation, as previously reported by the overexpression of GhSuSA1 and GhUGP1 under CaMV35S promoter (Jiang et al., 2012; Li et al., 2015).

Our cotton fiber analysis data clearly showed an increase in the length of the cotton fibers, from 26 mm in the non-transgenic control to 29.1 mm in the MA0023 transgenic cotton line (Figure 5). Similarly, the cellulose contents also increased, from 90% in the non-transgenic control cotton line to 96.2% in the MA0023 transgenic line (Figure 4). The results of cellulose correlates with previous studies where 95.88% and 97.7% cellulose are reported (Haigler et al., 2001; Wang et al., 2009). Considering the increased *SuS* gene expression and SuS enzymatic activity, it can be assumed that the increase in the secondary cellulose contents in the transgenic cotton lines occurred due to faster conversion of sucrose to other hexoses at the vegetative and fiber developmental stages (Pettogrew, 2001; Yuan et al., 2012). Increases in sucrose, at 15 DPA, and in total soluble sugars, at 30 and 40 DPA, were observed (Figures 3E,F), and these increases might influence the physiology of the cotton fibers through sucrose transport via a symplastic pathway (plasmodesmata), which may result in increased sucrose cleavage by activated SuS, as reported previously (Ruan et al., 2001, 2005, 2010). The fact that SuS expression was increased at 20 DPA in MA0023 and the other transgenic cotton plants (Figure 3B) suggests a role in fiber elongation. The trend of sucrose contents in transgenic and non-transgenic cotton fibers are in agreement with earlier studies (Jiang et al., 2012). The values of sucrose contents are lower than those reported previously (Pettogrew, 2001; Yuan et al., 2012). The variations in these sugar levels might occur on developmental, seasonal, diurnal, varietal, and environmental bases (Pettogrew, 2001; Yuan et al., 2012). The difference in fiber cellulose contents may be due to the change in *SuS* expression level and probably corresponds to difference in fiber quality (Yuan et al., 2012).

The SEM observations showed the increased fineness/smoothness and fiber crimp of some transgenic cotton plants in comparison to the non-transgenic control cotton plants (Figure 6). These changes may be due to the increased cellulose contents by elevated SuS catalytic activity that generated more hexoses leading to high cell turgor pressure that causes fiber elongation. The improvement in the surface fineness of the fiber surface is also supported by Haigler et al. (2001) that SuS increases carbon partitioning toward the cells that serve as a sink for deposition of cellulose during secondary wall synthesis. It can be assumed that the decrease in micronaire value in some transgenic plants is due to reduction in sucrose contents. Prior study also concluded that the decrease in fiber sucrose at the time of secondary wall synthesis perhaps contribute to the reduction of micronaire and extent of cellulose deposition in fiber ascertain the enhanced quality of fiber (Ryser, 1985; Delmer and Amor, 1995; Pettogrew, 2001; Martin and Haigler, 2004; Pan et al., 2010).

Morphological characteristics of plants were determined to note the effect of transgenic process on phenotypic features of plants. In comparison to non-transgenic control cotton plants, overexpression of SuS driven by CaMV35S promoter caused visible difference in phenotypic traits like plant height, boll number per plant, average boll weight, and leaf area (Figure 7). Overexpression of GhSuSA1 and GhUGP1 increased plant biomass, plant height, leaf area, and number of sympodial branches in cotton, *Arabidopsis*, and tobacco (Coleman et al., 2006; Wang et al., 2011; Jiang et al., 2012; Li et al., 2015). The results depict that transformation of SuS under CaMV35S promoter is expressed in whole cotton plant, and this expression is beneficial for vegetative growth and fiber characteristics as well.

Based on these results, we can infer that SuS is an essential enzyme involved in cotton fiber development, as the activity of this enzyme is correlated with cotton fiber quality. The study further concludes that there is a direct association between sucrose metabolism and fiber quality traits. Thus, *SuS* is a key target for improving the fiber characteristics and yield of local cotton cultivars to meet the demands of the mechanized textile industry. Furthermore, the transgenic cotton produced in this study is the best source for use in cotton variety development programs.

## Data Availability Statement

The datasets generated for this study are available on request to the corresponding author.

## Author Contributions

MA and AI conducted the experiments and wrote the manuscript. AL and SD helped in the practical work. AS and TH supervised and helped in the execution of the research work. AR conceived the idea, helped in the experimental design, and finalized the manuscript. XW and MBS contributed in the SEM and data analysis. All authors contributed to the article and approved the submitted version.

## Conflict of Interest

The authors declare that the research was conducted in the absence of any commercial or financial relationships that could be construed as a potential conflict of interest.

## Footnotes

1 https://www.ncbi.nlm.nih.gov

2 http://www.kazusa.or.jp/codon/

3 https://eu.idtdna.com/CodonOpt

4 https://web.expasy.org/translate

5 https://www.biobasic.com/gene-splash/

6 http://www.ccri.gov.pk

## Supplementary Material

Supplementary Figure 1 **(A)** Histochemical GUS staining of a cotton leaf. (1) Leaf from putative transgenic cotton plant showing blue-green color on leaf veins. (2) Leaf from a non-transgenic cotton plant used as a negative control without treatment with Agrobacterium. **(B)** PCR amplification of SuS gene from transgenic cotton plants. Lane 1-11: Amplification of 813 bp from experimental plants; Lane 12: Positive control (plasmid); Lane 13: Negative control (non-transgenic CEMB-00); M: 1 kb DNA ladder.

Supplementary Figure 2 Experimental design and generation advancement of SuS gene transgenic cotton plants from T0 to T1 in containment.

Supplementary Figure 3 PCR confirmation of T1 transgenic plants. Lane 1: Positive control (plasmid); Lane 2-5: Amplification of 813 bp from subtending leaves experimental plants; Lane 6: Negative control (non-transgenic CEMB-00); M: 1 kb DNA ladder.

Supplementary Table 1 Transformation efficiency of cotton embryos in used different experiments.
