# Supplementary material for: Overexpression of a Sucrose Synthase Gene Indirectly Improves Cotton Fiber Quality Through Sucrose Cleavage
Source: Front Plant Sci. 2020 Nov 12;11:476251. doi: 10.3389/fpls.2020.476251 (PMC7688987; doi:10.3389/fpls.2020.476251)
Supplement: Supplementary Figure 1 — (A) Histochemical GUS staining of a cotton leaf. (1) Leaf from putative transgenic cotton plant showing blue-green color on leaf veins. (2) Leaf from a non-transgenic cotton plant used as a negative control without treatment with Agrobacterium. (B) PCR amplification of SuS gene from transgenic cotton plants. Lane 1-11: Amplification of 813 bp from experimental plants; Lane 12: Positive control (plasmid); Lane 13: Negative control (non-transgenic CEMB-00); M: 1 kb DNA ladder. [file Image_1.pdf]

Supplementary Figures

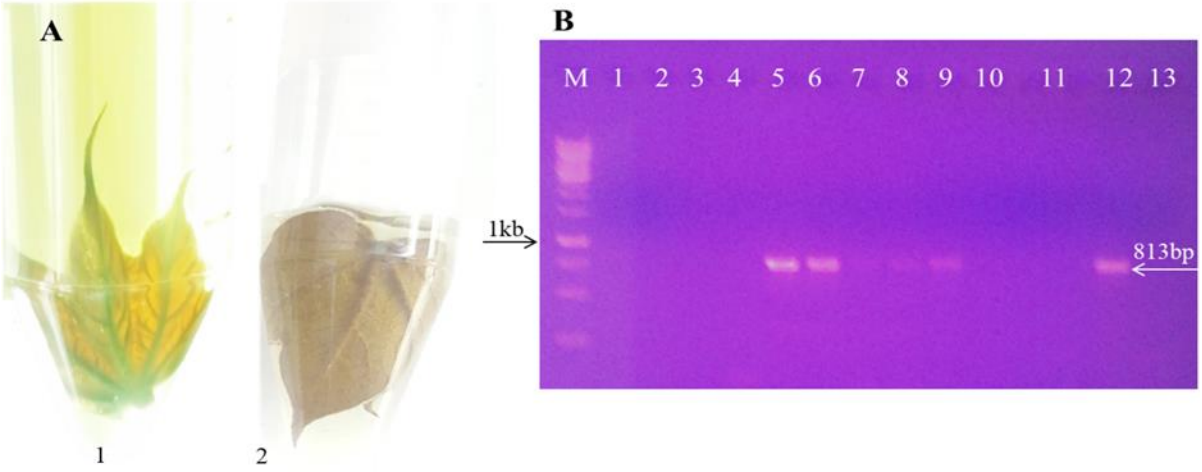

Figure S1 (A) Histochemical GUS staining of a cotton leaf. (1) Leaf from putative transgenic cotton plant showing blue-green color on leaf veins. (2) Leaf from a non-transgenic cotton plant used as a negative control without treatment with *Agrobacterium*. (B) **PCR amplification of *SuS* gene from transgenic cotton plants**. Lane 1-11: Amplification of 813 bp from experimental plants; Lane 12: Positive control (plasmid); Lane 13: Negative control (non-transgenic CEMB-00); M: 1 kb DNA ladder.

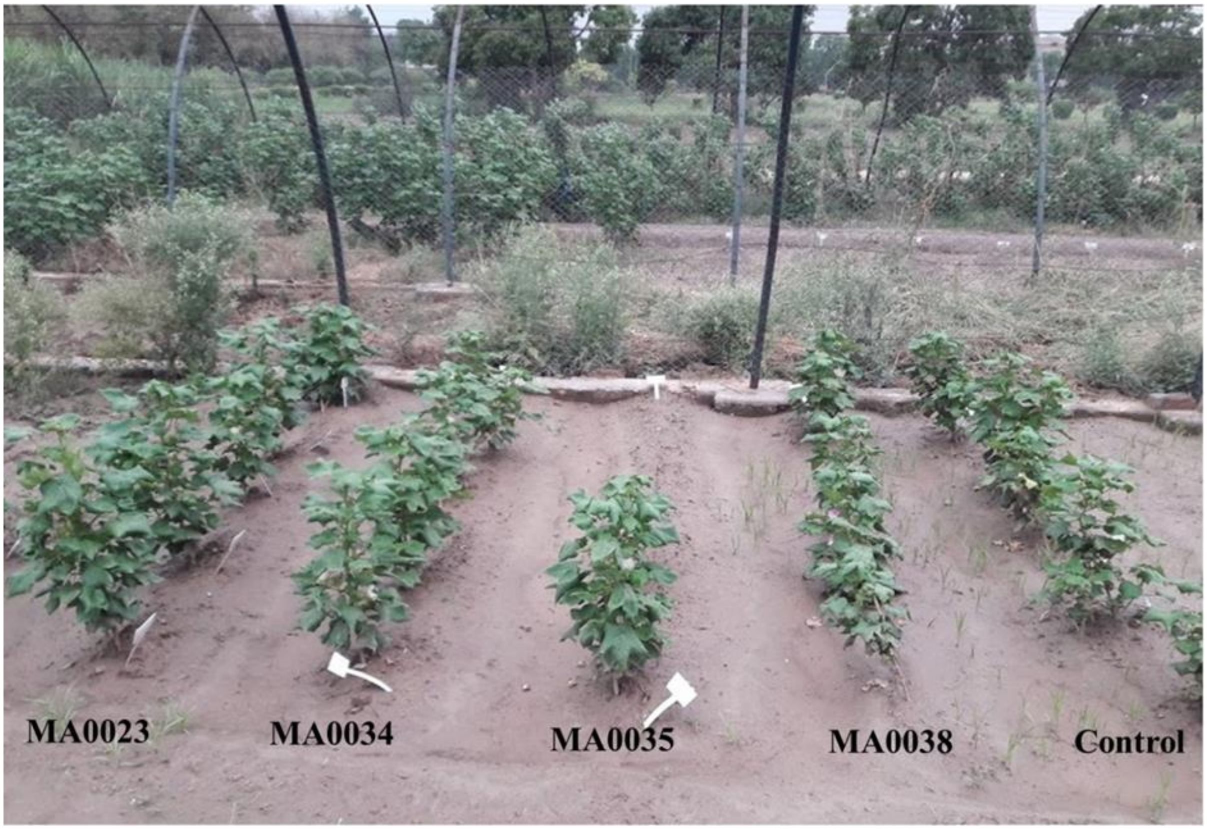

Figure S2 Experimental design and generation advancement of SuS gene transgenic cotton plants from T_0 to T_1 in containment.

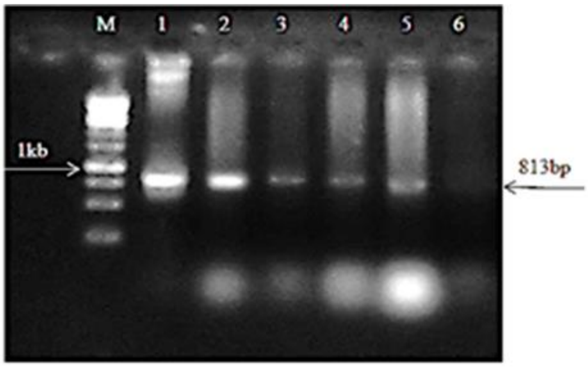

Figure S3 PCR confirmation of T_1 transgenic plants. Lane 1: Positive control (plasmid); Lane 2-5: Amplification of 813bp from subtending leaves experimental plants; Lane 6: Negative control (non-transgenic CEMB-00); M: 1kb DNA ladder.
