# Supplementary material for: Overexpression of a Sucrose Synthase Gene Indirectly Improves Cotton Fiber Quality Through Sucrose Cleavage
Source: Front Plant Sci. 2020 Nov 12;11:476251. doi: 10.3389/fpls.2020.476251 (PMC7688987; doi:10.3389/fpls.2020.476251)
Supplement: Supplementary Table 1 — Transformation efficiency of cotton embryos in used different experiments. [file Table_1.docx]

**Supplementary Table 1 Transformation efficiency of cotton embryos in used different experiments**

| **Experiment no** | **No of embryos isolated** | **Embryos treated with *Agrobacterium*** | **Control** | **No of embryos survived after 8 weeks** | | **No of plants shifted to pots** | **No of positive plants after PCR & GUS** | **TE %** |
| --- | --- | --- | --- | --- | --- | --- | --- | --- |
|  |  |  |  | Putative Transgenic | Control |  |  |  |
| 1 | 500 | 500 | 50 | 134 | 35 | 5 | 7 | 1.4 |
| 2 | 500 | 500 | 50 | 120 | 40 | 9 | 5 | 1.0 |
| 3 | 500 | 500 | 50 | 170 | 32 | 12 | 7 | 1.4 |
| 4 | 500 | 500 | 50 | 151 | 25 | 6 | 4 | 0.8 |
| 5 | 500 | 500 | 50 | 130 | 20 | 8 | 5 | 1.0 |
| 6 | 500 | 500 | 50 | 156 | 27 | 7 | 6 | 1.2 |
| 7 | 500 | 500 | 50 | 165 | 30 | 4 | 4 | 0.8 |
| 8 | 500 | 500 | 50 | 79 | 21 | 3 | 6 | 1.2 |
| 9 | 500 | 500 | 50 | 64 | 29 | 6 | 6 | 1.2 |
| 10 | 500 | 500 | 50 | 143 | 41 | 10 | 5 | 1.0 |
| 11 | 500 | 500 | 50 | 110 | 27 | 11 | 4 | 0.8 |
| 12 | 500 | 500 | 50 | 69 | 24 | 7 | 4 | 0.8 |
| 13 | 500 | 500 | 50 | 96 | 34 | 12 | 3 | 0.6 |
| 14 | 500 | 500 | 50 | 115 | 22 | 9 | 8 | 1.6 |
| 15 | 500 | 500 | 50 | 135 | 28 | 3 | 9 | 1.8 |
| 16 | 500 | 500 | 50 | 160 | 37 | 5 | 5 | 1.0 |
| 17 | 500 | 500 | 50 | 72 | 30 | 7 | 4 | 0.8 |
| 18 | 500 | 500 | 50 | 105 | 26 | 9 | 5 | 1.0 |
| Total | 9000 | 9000 | 900 | 2174 | 528 | 133 | 97 | 1.07 |
